# Nation-Wide Viral Sequence Analysis of HIV-1 Subtype B Epidemic in 2003–2012 Revealed a Contribution of Men Who Have Sex With Men to the Transmission Cluster Formation and Growth in Japan

**DOI:** 10.3389/frph.2020.531212

**Published:** 2020-12-03

**Authors:** Teiichiro Shiino, Atsuko Hachiya, Junko Hattori, Wataru Sugiura, Kazuhisa Yoshimura

**Affiliations:** ^1^Surveillance and Information Division, Infectious Disease Surveillance Center, National Institute of Infectious Diseases, Tokyo, Japan; ^2^Division of Biological Information Analysis, Department of Clinical Research Management, Crinical Research Center, National Hospital Organization Nagoya Medical Center, Nagoya, Japan; ^3^AIDS Research Center, National Institute of Infectious Diseases, Tokyo, Japan; ^4^Research Institute Director, Tokyo Metropolitan Institute of Public Health, Tokyo, Japan

**Keywords:** HIV-1, subtype B, transmission cluster, risk population, MSM, phylogenetic analysis

## Abstract

**Background:** To better understand the epidemiology of human immunodeficiency virus type 1 (HIV-1) subtype B transmission in Japan, phylodynamic analysis of viral *pol* sequences was conducted on individuals newly diagnosed as HIV-1 seropositive.

**Methodology:** A total of 5,018 patients newly diagnosed with HIV-1 infection and registered in the Japanese Drug Resistance HIV Surveillance Network from 2003 to 2012 were enrolled in the analysis. Using the protease-reverse transcriptase nucleotide sequences, their subtypes were determined, and phylogenetic relationships among subtype B sequences were inferred using three different methods: distance-matrix, maximum likelihood, and Bayesian Markov chain Monte Carlo. Domestically spread transmission clusters (dTCs) were identified based on the following criteria: >95% in interior branch test, >95% in Bayesian posterior probability and <10% in depth-first searches for sub-tree partitions. The association between dTC affiliation and individuals' demographics was analyzed using univariate and multivariate analyses.

**Results:** Among the cases enrolled in the analysis, 4,398 (87.6%) were classified as subtype B. Many of them were Japanese men who had sex with men (MSM), and 3,708 (84.3%) belonged to any of 312 dTCs. Among these dTCs, 243 (77.9%) were small clusters with <10 individuals, and the largest cluster consisted of 256 individuals. Most dTCs had median time of the most recent common ancestor between 1995 and 2005, suggesting that subtype B infection was spread among MSMs in the second half of the 1990s. Interestingly, many dTCs occurred within geographical regions. Comparing with singleton cases, TCs included more MSM, young person, and individuals with high CD4+ T-cell count at the first consultation. Furthermore, dTC size was significantly correlated with gender, age, transmission risks, recent diagnosis and relative population size of the region mainly distributed.

**Conclusions:** Our study clarified that major key population of HIV-1 subtype B epidemic in Japan is local MSM groups. The study suggests that HIV-1 subtype B spread via episodic introductions into the local MSM groups, some of the viruses spread to multiple regions. Many cases in dTC were diagnosed during the early phase of infection, suggesting their awareness to HIV risks.

## Introduction

Identifying geographical and/or temporal prevalence of a viral transmission, the transmission cluster (TC), gives important information in preventing the spread of infections. Spatio-temporal statistical analyses (1, 2) are generally used to identify a disease cluster, which may not directly represent the transmission of an infectious agent. Another approach in identifying TC is through social network surveillance using aggressive field epidemiological surveillance. However, this is ethically difficult in HIV/Acquired Immunodeficiency Syndrome (AIDS) because of privacy issues. Viral sequence-based inference of TCs is a realistic and ideal alternative method to social network surveillance, elucidating an accumulation of HIV transmission events at a local region. Recent progress in bioinformatics allows more detail of such kind of study.

In Japan, HIV/AIDS registration system started in 1984, requiring physicians to report all diagnosed cases. Since the first HIV-1 infected case in Japan reported in 1985, the total number of reported cases has been increasing annually, reaching to 27,443 at the end of 2017 (3). Approximately 30% represent AIDS cases at diagnosis, and the proportion has not been decreased over the last decade, suggesting the need of more effective preventive measures. Since 73% of newly reported cases were associated with men who had sex with men (MSM) including bisexual contacts (3), MSM is the key population in Japan like many other high- and middle-income countries (4, 5). Thus, to decrease the number of persons who do not know their HIV status in Japan, we need to know HIV transmission dynamic in the MSM key population. Although most Japanese sero-positive MSMs were known to be infected with HIV-1 subtype B (6, 7), how subtype B viruses were introduced and spread among MSM population in Japan remains to be elucidatedC:\Users\tshiino\Documents\GetARef\Refs\myref.ref #131.

We have been collecting viral nucleotide sequence data from newly diagnosed individuals as the Japanese Drug Resistance HIV Surveillance Network (6, 8, 9), which covers more than 40% of HIV-1 cases officially reported in Japan. This allowed us to estimate nation-wide transmission dynamics of the subtype B virus in Japan using phylodynamic approach to the large-scale sequence and demographic information from the surveillance. In this paper we identified subtype B viruses were simultaneously spread in >300 domestically expanding TCs, which were introduced in Japan from the 1990's to 2000's. TCs with many individuals, which were episodic transmitting groups of people living with HIV/AIDS (PLWHA), were composed of MSM. Large TCs consisted of individuals in the same geographic region, of young age and who were diagnosed early. These results provide epidemiological information which may support preventive strategies involving public health interventions to reduce new HIV transmissions in key populations in Japan.

## Materials and Methods

### Ethics Statement

This study was conducted according to the principles in the Declaration of Helsinki. The study was approved by the human subject research committee at the National Institute of Infectious Diseases and National Hospital Organization Nagoya Medical Center, Japan (Approved no. 2010-310). All patients provided written informed consent for the collection of samples and subsequent analyses.

### Study Subjects

**Figure 1:**
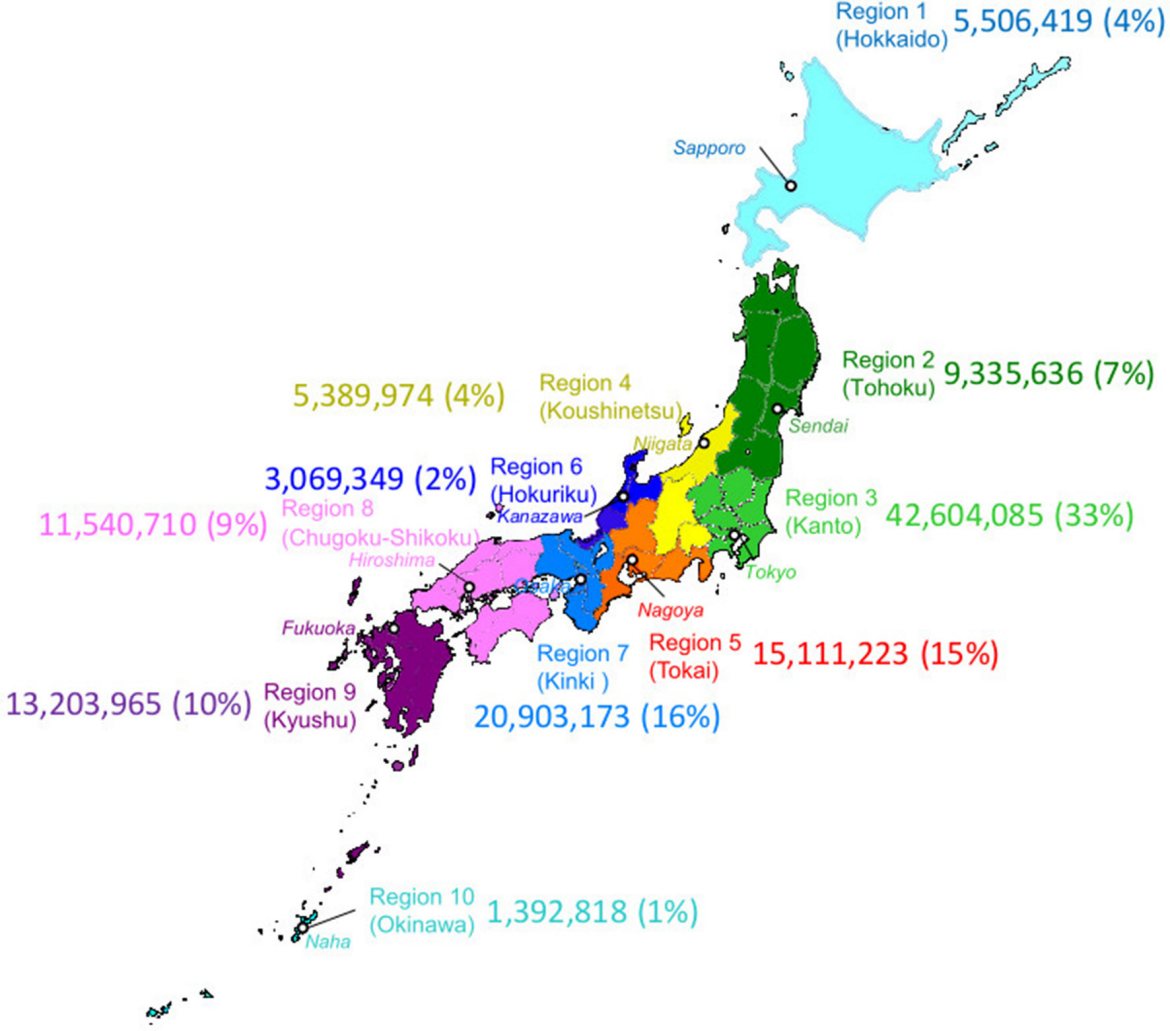
Geographic map of administrative regions in Japan. Ten regions in Japan are designated by the same colors as used in other figures to indicate the region diagnosed. The major city in a region were indicated in a white circle with italicized name. Numbers indicated population and regional population ratio with the total population in Japan (PopRatio) of each region in 2010 Population Census (by Statistics Bureau, Japan. http://www.stat.go.jp/data/kokusei/2010/kihon1/pdf/gaiyou2.pdf); (https://www.freemap.jp).

The Japanese Drug Resistance HIV Surveillance Network (6, 8, 9) enrolled 5,922 newly diagnosed and ART naïve PLWHAs between January 2003 and December 2012 at 30 clinics and public health centers located in one of the ten administrative regions in Japan (Figure 1). The patient's peripheral blood was drawn into a vacutainer with EDTA added at diagnosis or the earliest hospital visit. The CD4+ T-cell count and the HIV viral load (VL) were also collected for the surveillance where available. Among these cases, 5,018 possessing the nucleotide sequence information of HIV-1 protease (PR: 297bp) and the 1- to 240-amino acid region of reverse-transcriptase (RT: 720bp) (HXB2:2253-3269) using the direct sequencing method of RT-PCR products from the patients' plasma samples were subjected to the following analyses. The detailed methods for CD4+ T-cell count, VL, RT-PCR and nucleotide sequencing were described in the previous reports (6, 8–10).

### Subtype Identification and Selection of Subtype B-Infected Cases

Nucleotide sequences of PR and RT genes were concatenated and aligned with subtype reference alignment set of HIV-1 M group with CRF01_AE, CRF02_AG, CRF07_BC, and CRF08_BC in the Los Alamos HIV database (http://www.hiv.lanl.gov/content/sequence/NEWALIGN/align.html) using MUSCLE (11) built in MEGA7 (12). Subtypes were determined by this alignment using similarity plot analysis against subtype reference by estimating the number of substitution using Tamura-Nei (TN93) model (13) by in-house program written in Perl5, and 4,398 samples of entirely subtype B were selected for the further analyses.

### Phylodynamic Analysis for Identification of Domestically Spread TCs (dTCs)

**Figure 2:**
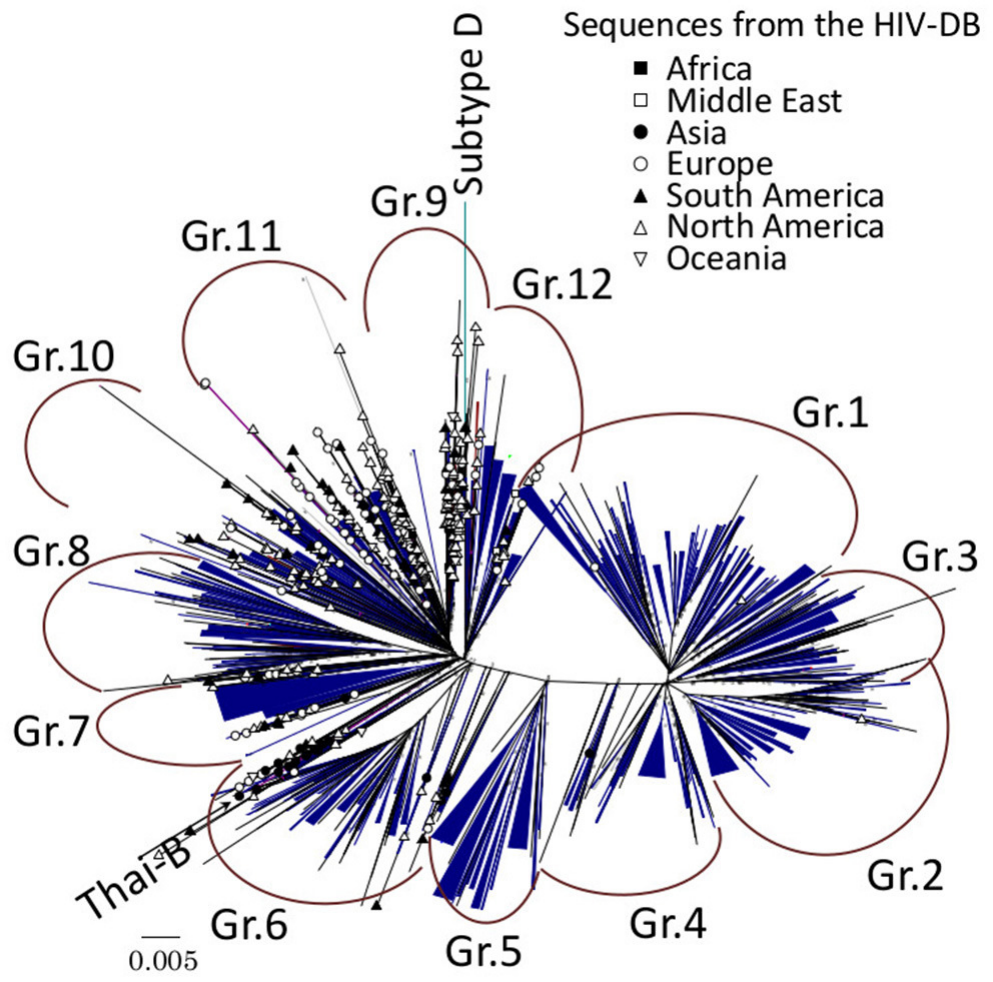
Maximum-likelihood (ML) phylogeny of subtype B sequences from the HIV drug resistance surveillance study in Japan. A total of 4,398 subtype B sequences from the enrolled cases and 644 subtype B reference sequences from Los Alamos HIV database (HIV-DB) were used for the analysis, and 312 dTCs were identified. Each dTC is simplified in blue triangles in ML tree with unrooted radial form. Reference sequences are designated by symbols representing the country of origin.

We define dTCs as a monophyletic group without paraphyletic foreign outlier, and sharing less diversified viral sequences as described in the previous report (10). First, a primary guide tree was constructed using 4,398 subtype B cases which determined 12 phylogenetically divergent groups (Gr.1 to Gr.12 in Figure 2). Second, in order to detect an ancestral node of each case introduced in Japan in the phylogeny, we selected a representative sequence from each group and searched the Los Alamos HIV database for the similar references of the group representative using BLAST and yielded top 100 similar entries. From them, we excluded entries without collection years and collected in Japan, and finally obtained 51–82 reference sequences which did not diverge from the group. The total number of reference sequences was 644 due to duplications of entries between groups. Reference sequences were re-aligned with the sample sequence and four subtype D outliers, 01CM_4412HAL, 94UG114, A280, and ELI. Then, ambiguous loci containing multiple peaks of nucleotides were converted to a plausible one using the method described in the previous report (10). To eliminate the influence of antiretroviral drug treatments on viral evolution, we removed 43 drug resistance-associated codons defined in the previous studies (6, 8). From those alignments, we constructed phylogenies inferred by 3 different methods: neighbor-joining (NJ) method, maximum likelihood (ML) method, and Bayesian Markov chain Monte Carlo method. According to the results of the interior branch test (14) with 1,000 bootstrap resampling (15), Felsenstein's bootstrap test (16) with 1,000 resampling, or posterior probability of Bayesian analysis (17), we searched in depth-first manner for significant monophyletic groups including sample sequences from the phylogeny. In this step, a paraphyletic overlapping the foreign reference sequence was regarded as a splitter except for singleton or cluster of foreign reference sequence(s) without a significant branch. NJ, ML, and substitution model selection were conducted using MEGA7 (12). Bayesian maximum clade credibility (BMCC) chronological trees were inferred using BEAST 1.8 (18). According to the model selection (Supplementary Table 2), we applied TN93 and the general time reversible model with gamma-distributed site heterogeneity and invariant sites (GTR+G+I) to infer NJ and ML or BMCC trees, respectively. To evaluate the diversity-based criterion, we measured median sub-tree nucleotide diversity of each cluster candidate as described in the previous reports (10, 19). Eventually, only the monophyletic groups fulfilling all those criteria were recognized as dTCs. Median time of the most recent common ancestor (tMRCA) were also inferred using BEAST 1.8 with the exact date of samples collected. Models for the clock and population were evaluated in each group by log marginal likelihood estimation using path sampling and stepping-stone sampling (20, 21) in BEAST 1.8 (Supplementary Table 3).

### BED Assay

Whether the HIV-1 seroconversion occurred recently (within 6 months) was estimated using the BED IgG-Capture Enzyme immunoassay (BED assay, Calypta HIV-1 BED Incidence EIA, BioRad) as described previously (6, 9). To reduce overestimation of the recent incidence (22), we excluded the cases having <50 CD4+ T cells/μL or HIV RNA level of <1,000 copies/ml. Briefly, 101× diluted plasma samples were used to microplate wells in the kit and prepared according to the manufacturer's instructions. To obtain normalized optimal density values (ODn), each reading at 450 nm wavelength was validated using controls and calibration to decrease run-to-run variability. Cases with ODn of ≤0.8 were interpreted as “recent” seroconverters and ODn of >0.8 as “not recent” seroconverters.

### Statistical Analysis

Association between demographic characteristics and dTCs was tested by Fisher's exact probability. Multivariate logistic regression analysis was performed for four parametric variables, “age,” “regional population ratio with the total population in Japan (PopRatio: see Figure 1),” “CD4+ cell count,” and “VL,” on individuals into dTC. Association between parametric and categorical variables of each case and the dTC was analyzed by multivariate logistic regression and multiple regression analyses using dummy variables by Hayashi's quantification method type I (23), respectively. Difference on CD4+ T-cell counts or viral RNA levels of each sample between dTC and singleton was assessed using Mann-Whitney U test. Trends in the geographical distribution of each dTC were analyzed by hierarchical clustering Ward's method. All statistical analyses were calculated using R version 3.4.1.

## Results

### The Majority of Cases Were Japanese MSM Infected With Subtype B HIV-1

Among the 5,018 cases collected from 2003 to 2012, 4,398 (87.6%) were determined as subtype B by phylogenetic analysis. Remaining 620 cases were classified into CRF01_AE (*n* = 358: 7.1%), C (*n* = 49), CRF02_AG (*n* = 29), G (*n* = 22), F (*n* = 9), CRF06_cpx (*n* = 3), CRF07_BC (*n* = 2), CRF12_BF (*n* = 2), CRF33_01B (*n* = 2), D (*n* = 1), CRF08_BC (*n* = 1), and CRF28/29_BF (*n* = 1). There were 141 unclassified cases which did not match with any of the known subtype/CRF patterns, suggesting unidentified intra-subtype recombinants.

Of 4,398 subtype B-infected individuals, 65.2% reported male to male and/or bisexual sexual contacts (Supplementary Table 1). While majority were Japanese cases among the cases where nationality is obvious (96.2%), 167 individuals originated from other countries, where Asian (*n* = 54: 1.23%) and South American (*n* = 47: 1.07%) countries were prevalent. Only 60 individuals were women (1.4%), and the number of men with heterosexual risk (*n* = 381) was 7-fold higher than that of women (*n* = 54). The median age of individuals at diagnosis was 36.05 years with 29.9 and 43.1 years as the first and the third quartiles, respectively. Majority of collected cases (*n* = 3,904; 88.8%) were reported from any of three metropolitan areas in Japan, Kanto, Kinki or Tokai region. No demographical differences were observed according to gender, risk factor and nationality among the three regions.

### 312 Domestically Spread TCs Were Determined

**Figure 3:**
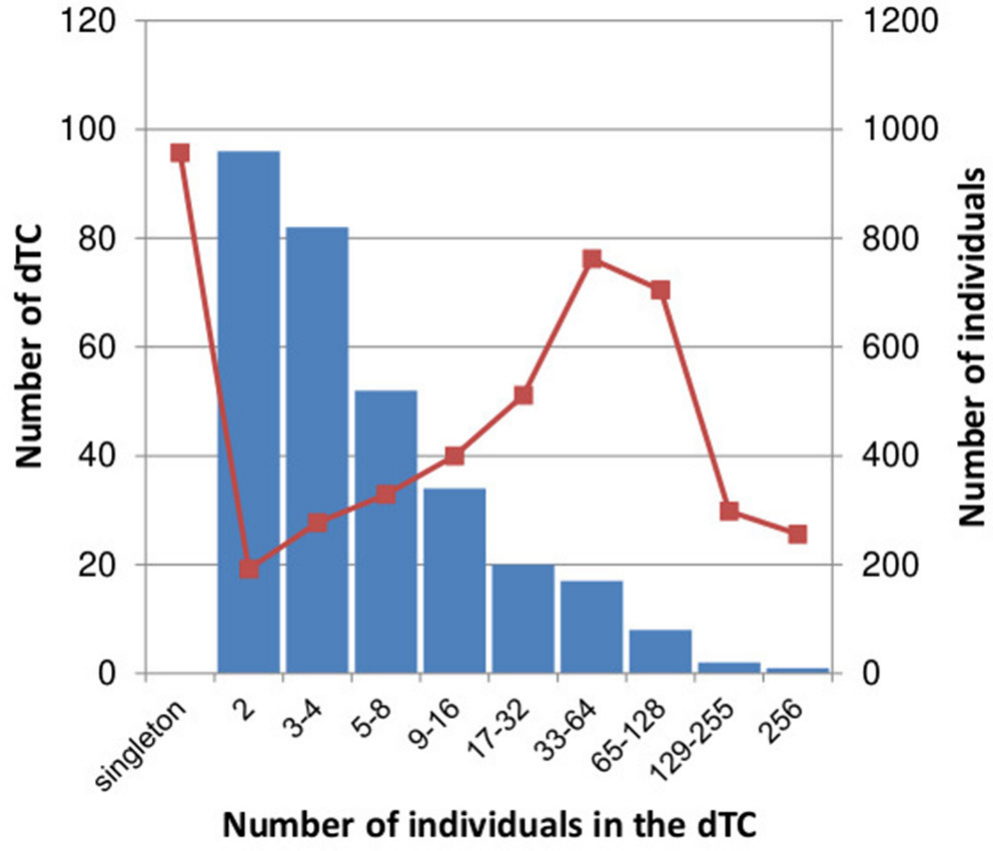
Distribution of the number of dTC and total number of individuals in each dTC size. The number of dTCs in each range of dTC size is represented in blue bar. The total number of individuals in dTCs within each range is represented in red square with line.

Of 4,398 subtype B cases, 3,714 (84.4%) were clustered into 312 dTCs with at least one potential partner (Figure 2), whereas 684 remaining cases did not belong to any dTCs, i.e., singletons. dTCs were numbered according to the descending number of cases. A distribution of the size and frequencies of dTC is summarized in Figure 3, where the dTCs including ≥20 individuals (dTC_≥20_) was 44 (14% of total number into dTCs), and the largest one (TC1) included 256 individuals. Total number of individuals included in all dTCs_≥20_ were 2,441, being 55.5% of the total population (Figure 3). The number of the individuals included for each TC size range showed a bell-shaped distribution with a maximum at 32–63 (*n* = 762) (Figure 3).

### MSM Was the Major Transmission Risk in dTC_≥_20

**Figure 4:**
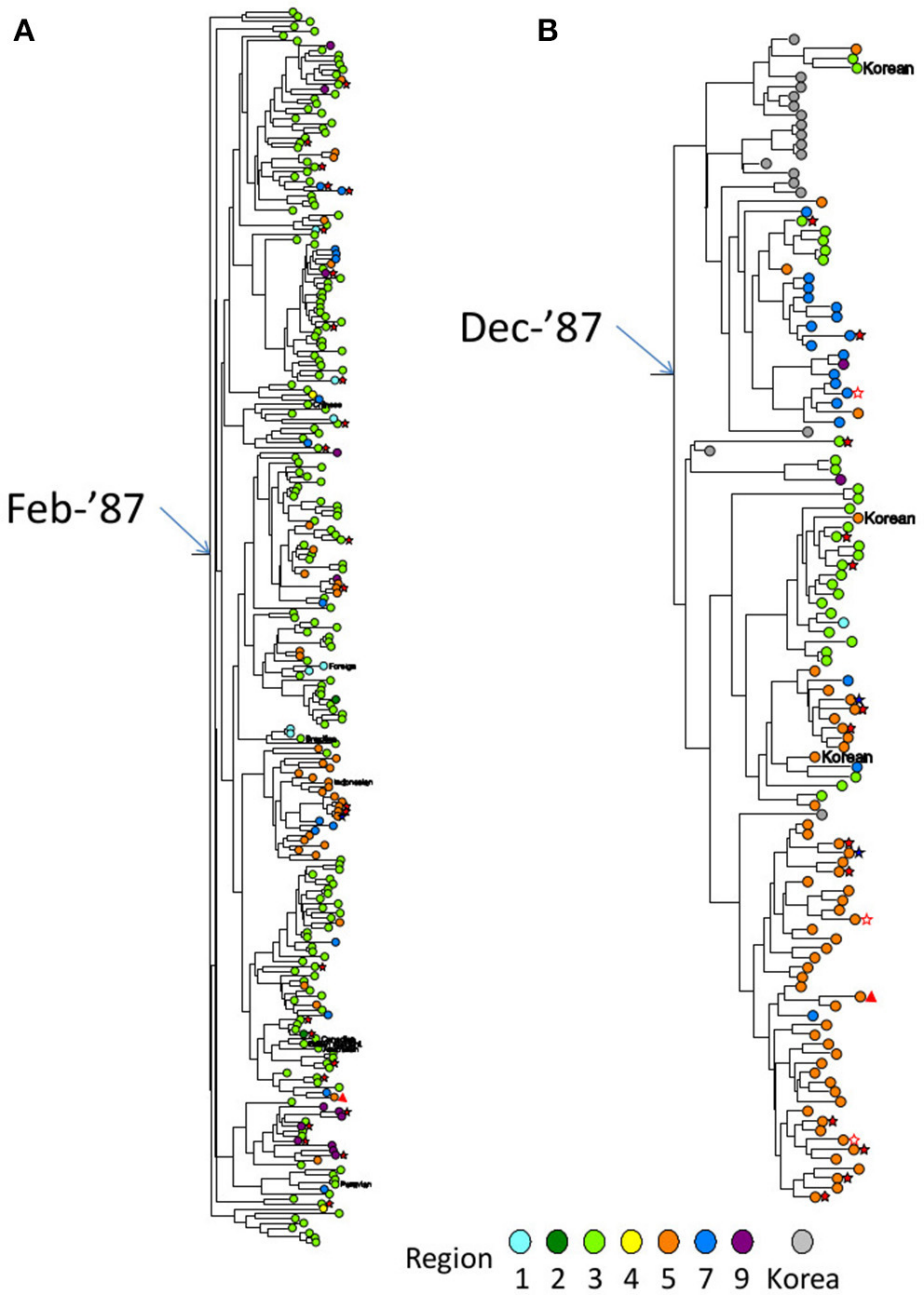
Partial Bayesian clade credibility tree of two extremely large dTCs identified in Japan. **(A)** The monophyletic group constituting TC001, which was the largest dTC in Japan. **(B)** The monophyletic group constituting TC004, which was the 4th largest dTC with Korean foreign references in ancestral nodes. Maximum clade-credibility chronological tree was inferred by Bayesian MCMC method using BEAST 1.8. Clock and demographic model adopted for the inferences were strict clock/constant size and exponential/constant size for TC001 and TC004, respectively. Sequences from subjects are designated by circles colored representing their regions diagnosed. Gray circle shows the reference sequence from Korea. Transmission risk declared by the individual is shown in symbols as follows: MSM as no mark, bisexual male as blue five-pointed star, filled heterosexual male as red five-pointed star, heterosexual female as open five-pointed star and IDU as triangle. Non-Japanese individuals' nationalities are represented by red circles with their country code as same as the HIV database (https://www.hiv.lanl.gov/content/sequence/HelpDocs/databasecountrycode.html).

Approximately 60 and 45% of cases with MSM-declared individuals were involved in dTCs and the singleton group, respectively (Table 1A). Furthermore, 97.6% (3,625) of dTC affiliating cases were men while 68.8% in the singletons. Figures 4A,B showed phylogenetic trees of two large dTCs with characteristics, TC001 being the largest one, and TC004 originating from individuals in Korea. The trees included both subclusters consisting of the genetically closely related viruses with the similar collection years and parts where the viruses from different collection years are connected by long branches. Many of the other dTCs showed the same trend. Although some dTC_≥20_ included individuals who reported their risks as heterosexual contacts, phylogenetic analysis showed that their viruses were closely related to that of MSM and no potential female partners were found (Figure 4). Since dTC_≥20_ contained a few foreign and/or non-sexual contact cases, such individuals were not the key player to structure the dTC_≥20_. In contrast, non-MSM risks were occasionally observed in small dTCs. Indeed, heterosexual contact was estimated as the major risk in six dTCs (*n* = 5, 4, and four pairs), and intravenous drug usage was identified in one female pair. Thus, dTC analysis clarified that the major transmission risk of subtype B-infected patients constituting dTC_≥20_ in Japan were Japanese MSMs including bisexuality.

**Table 1A:** Analysis of associating demographic and clinical characteristics with the affiliation of the large TCs (≥20).

	**Clustered**	**Unclustered**	**Total**	**Clustered rate %**	**Odds ratio**	**95% C.I**.	***P*-value**	
**(A) THE UNIVARIATE CONTINGENCY ANALYSIS BY FISHER'S EXACT TEST**								
Total number of individuals	3,708	678	4,386	84.5				
**Gender**								
Male	3,625	644	4,269	84.9	3.030	1.635–5.171	0.0002	^***^
Female	39	21	60	65.0				
Unknown	44	13	57	77.2				
**Transmission risks**								
MSM or bisexual	2,456	436	2,892	84.9	1.564	1.206–2.017	0.0060	^**^
Heterosexual	342	95	437	78.3				
IVDU or unidentified	910	147	1,057	86.1				
**Nationality**								
Japanese	2,977	509	3,486	85.4	1.448	0.895–2.269	0.1029	
Foreigner	105	26	131	80.2				
Unidentified	626	143	769	81.4				
**Age**								
<40 y.o.	2,377	307	2684	88.6	1.927	1.585–2.339	<10^−11^	^***^
≥40 y.o.	876	218	1,094	80.1				
Unidentified	462	349	811	57.0				
**BED assay**								
Recent	713	78	791	90.1	1.466	1.105–1.958	0.0064	^**^
Not recent	1,297	208	1,505	86.2				
Unexamined	1,698	392	2,090	81.2				

*** *Significance level p < 0.001*,

** *Significance level p < 0.01*.

### dTC_≥_20 Showed the Region Specificity

**Figure 5:**
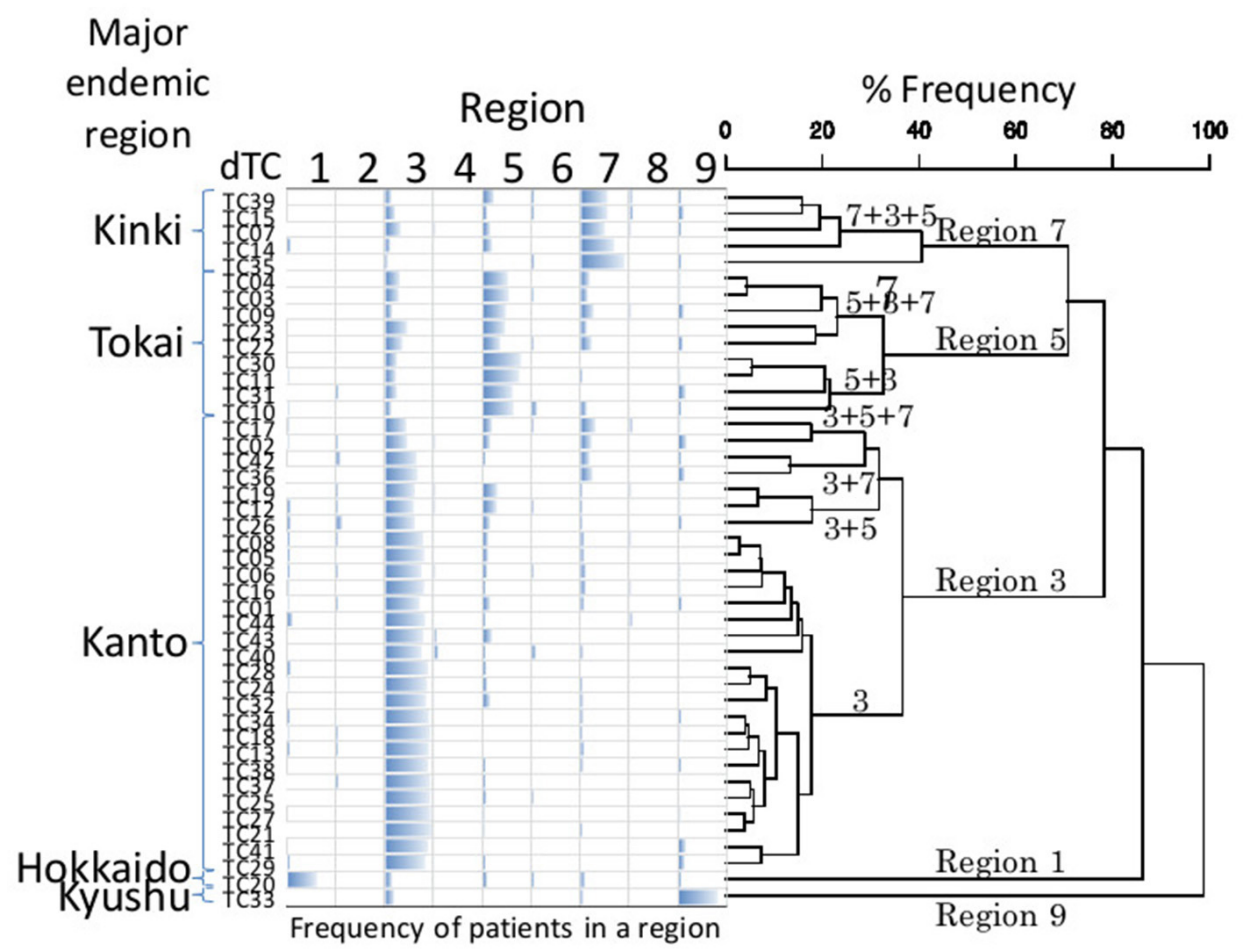
UPGMA cluster analysis of geographic property of dTC. Bar chart represents the frequency of individuals belonging to a dTC according to a region. The order of dTCs in the chart is decided by cluster analysis using UPGMA. The numbers above a branch show major regions that a member of dTC is derived. Refer Figure 1 for the correspondence between the numbers and the regions. Each UPGMA group (geographic category) is named by a composition of the major regions.

**Figure 6:**
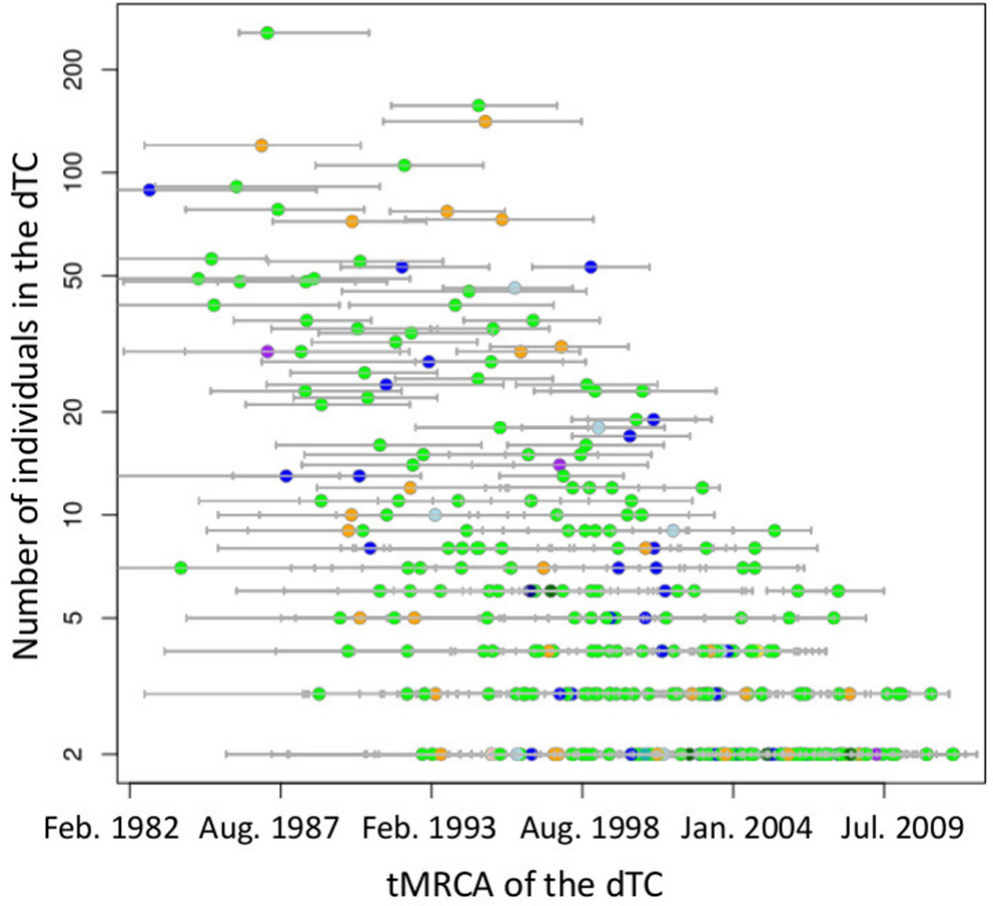
Distribution of tMRCA of subtype B TCs on the size of dTC. Small circles indicate TCs, color coded by diagnosed region according to Figure 1. Bars on the circle show 95% highest posterior density interval of tMRCA.

According to their geographic region of diagnosis, dTC_≥20_ can be classified into five categories (Figure 5). Each category shared a most prevalent region of the infection, i.e., 28 dTC_≥20_ in Kanto (Region 3), 9 dTC_≥20_ in Tokai (Region 5), 5 dTC_≥20_ in Kinki (Region 7), 1 dTC_≥20_ in Hokkaido (Region 1), and 1 dTC_≥20_ in Kyushu (Region 9). dTC_≥20_ made some sub-clusters among the categories depending on the second and third prevalence. These region specificities demonstrated the presence of major endemic regions where the virus was transmitted locally. Most of dTC_≥20_ had tMRCA between the late 1980's to early 1990's, which did not differ significantly among the major endemic regions of dTC_≥20_ (Figure 6). In contrast, few small dTCs and no pair cases had tMRCA before 1990.

dTC tended to include early diagnosed cases in young and sexually infected male from urban region were consistent of young and early staged cases

**Figure 7:**
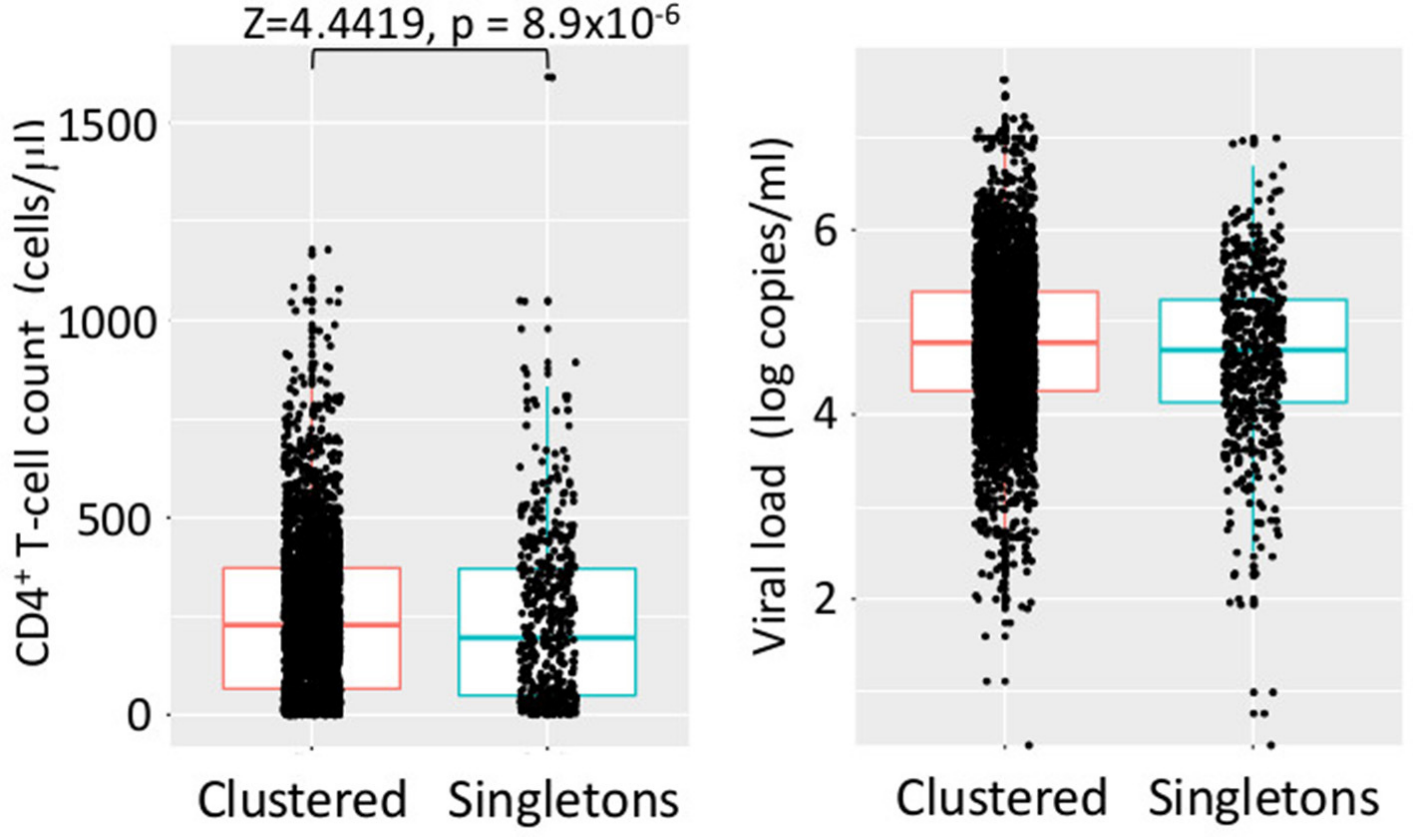
Distribution of CD4^+^ T-cell counts and VLs of individuals in dTCs and singletons. Dots indicate clinical values of individual at diagnosis. In every case, H_0_ (normal distribution) was rejected by Shapiro–Wilk test (*p* < 10^−15^). Significant results of statistical test for differences in clustered and singleton cases are shown in a bracket.

**Figure 8:**
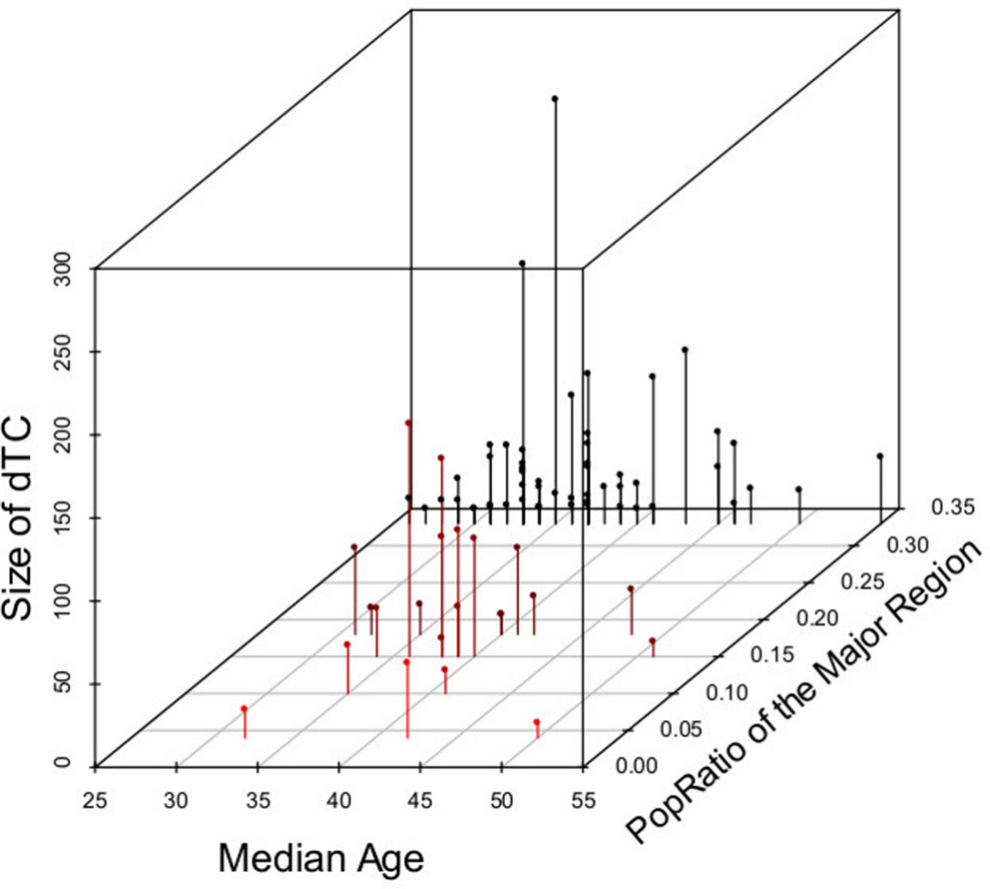
Relationship across dTC size, PopRatio of the main distributed region, and median age in dTCs_≥10_. Dots indicate the ratio of regional population to total population in Japan (PopRatio), the median age, and size of dTCs_≥10_. Each dot is colored from black to red according to the PopRatio”.

Cases in dTC included younger and recently diagnosed individuals compare to singleton cases (Table 1A). Individuals' age and the size of the dTC demonstrated a positive correlation in semi-log linear regression analysis (Adj. *R*^2^ = 0.002, F = 10.17, *p* = 0.001). CD4+ T-cell count distribution in cases clustered into dTC slightly drifted to higher levels compared to that of singletons (*p* = 8.9 × 10^−6^, Figure 7). However, the frequency of individuals diagnosed with advanced disease (CD4+ T-cell count <400 and/or VL >10^4^) did not correlate with the dTC sizes. In univariate analysis, clustered cases were more likely to be men, MSM, or bisexual, and recently diagnosed, but were not associated with their nationality (Table 1A). In multivariate analysis, in which (Age*PopRatio)+CD4+VL was recommended as the best logistic regression model by Akaike's Information Criterion (AIC) in both ≥10 (dTC_≥10_) and ≥20 (dTC_≥20_) thresholds of dTC size (Supplementary Table 3), Age and PopRatio were negatively correlated in dTC_≥10_ (Table 1B), i.e., the dTC size was inversely correlated with age at a particular size of city. Indeed, Figure 8 shows that dTC with a low median age tended to have a large size in the middle PopRatio (shown in blown dots) but not in the Kanto region (black dots). Interaction between “age” and “reginal population size” was marginally significant (*p* = 0.077), suggesting individuals in Kanto and Kinki, which are densely populated regions, tended to be young. “VL” positively correlated with the property of dTC affiliation in both dTC_≥10_ and dTC_≥20_, although the regression coefficients were low (beta = 1.48 × 10^−7^ and 1.51 × 10^−7^, respectively). However, “CD4+ T-cell count” and “VL” did not correlate with dTC affiliations unlike the univariate test. We also performed correlation analysis on five categorical variables by converting those with dummy variables (Table 2). In gender, women were negatively associated with dTC_≥20_ affiliation. In the collected region, Tokai (PopRatio = 15%) and Kyushu (PopRatio = 10%) regions were positively, and Chugoku-Shikoku (PopRatio=9%), Hokkaido (PopRatio = 4%), Koushinetsu (PopRatio = 4%), Tohoku (PopRatio = 7%), and Okinawa (PopRatio = 1%) regions were negatively associated with dTC_≥20_ affiliation. Non-sexual contacts (blood transfer, blood product, IDU and MCT in Table 2) were negatively associated with dTC_≥20_ affiliation. The BED assay and the nationality were analyzed separately because these surveys were conducted in a limited number of cases. The result showed that the number of early diagnosed individuals tended to be higher in dTC_≥20_.

**Table 1B:** Multivariate logistic regression test.

**Term**	**TC size**	**b estimate**	**Standard error**	***z*-value**	**Adjusted OR (95% C.I.)**	***P*-value**	
(Intercept)	≥10	1.860	0.222	8.369	3.348 (1.860–10.71)	<0.0001	^***^
	≥20	0.870	0.455	1.912	1.912 (0.978–5.827)	0.056	
Age	≥10	−0.011	3.95 × 10^−3^	−2.687	0.998 (0.978–1.020)	0.007	^**^
	≥20	0.012	0.011	1.100	1.012 (0.991–1.035)	0.271	
Regional population ratio	≥10	−1.690	0.540	−3.133	1.039 (0.025–43.22)	0.002	^**^
	≥20	1.260	1.940	0.648	3.515 (0.079–157.2)	0.517	
CD4^+^ T-cell count	≥10	−1.38 × 10^−4^	2.29 × 10^−4^	−0.601	0.999 (0.999–1.000)	0.548	
	≥20	4.95 × 10^−5^	2.35 × 10^−4^	0.211	1.000 (0.999–1.001)	0.832	
Viral load	≥10	1.48 × 10^−7^	5.71 × 10^−8^	2.593	1.027 (0.995–1.048)	0.090	
	≥20	1.51 × 10^−7^	5.63 × 10^−8^	2.679	1.076 (1.000–1.118)	0.070	
Age: regional population ratio	≥10	−0.086	0.049	−1.768	0.956 (0.871–1.049)	0.077	
	≥20	−8.58 × 10^−2^	0.049	−1.768	0.918 (0.835–1.009)	0.077	

*OR, Adjusted odds ratio*,

*** *Significance level p < 0.001*,

** *Significance level p < 0.01*.

**Table 2:** Categorical variate analysis of demographic characteristics associating with the size of TCs using Hayashi's quantification theory type I.

**Variates**	**Category**	**Category score**	**Partial corr. coeff**.	***t*-value**	***P*-value**
Gender	Male	0.452	0.054	3.553	<0.001
	Female	−29.183			
	Unknown	−13.462			
Region	Kanto (33%)	−3.494	0.128	8.581	<0.001
	Kinki (16%)	−3.576			
	Tokai (15%)	17.729			
	Kyushu (10%)	6.190			
	Chugoku-Shikoku	−15.910			
	(9%)				
	Tohoku (7%)	−16.035			
	Koushinetsu (4%)	−12.721			
	Hokkaido (4%)	−8.664			
	Hokuriku (2%)	−3.178			
	Okinawa (1%)	−46.241			
Risk	MSM	0.671	0.046	3.057	0.002
	Heterosexual	1.580			
	Bisexual	−13.288			
	Blood transfer	−34.076			
	Blood product	−47.976			
	IDU	−10.365			
	MCT	−48.076			
	Unidentified	−1.126			
Constant		52.118			
Nationality^*^	Japanese	−0.033	0.002	0.079	0.937
	Other countries	0.648			
Result of BED^*^	Recent	2.270	0.022	0.859	0.391
	Not recent	−1.120			
constant^*^		59.289			

* *excluded cases without BED assay (total of 1,462 individuals)*.

## Discussion

Since our drug resistance surveillance network recruited the high coverage of PLWHA from all over Japan, and the method and reference sequences were carefully selected to identify domestically limited transmissions, the results shown in this study will represent the trends of nation-wide subtype B epidemic in Japan. Subtype B viruses were introduced in Japan through hundreds of different origins between the 1990's and 2000's and then each spread in episodic manner. TC-affiliating ratio of subtype B in Japan (3,714/4,398: 84.4%) was significantly higher than that of CRF01_AE in Japan (31.3% included in 30 clusters) (10) as well as subtype B in London (23.7%) (24) and Washington DC (13.5%) (25), suggesting that the domestic transmission event of subtype B virus was extremely frequent in Japan. Actually, we found several large dTCs involving >100 PLWHAs. Shape of the size distribution, as presented here (Figure 3), was right-hand tailed, as in the London study (24) and CRF01_AE (10); however, its decrease was more gradual, suggesting its tendency to create a domestic cluster, relative to those cases. These results raise public health concerns that subtype B viruses are more likely to spread in the domestic at-risk population.

Our finding indicated that MSM was the key population for subtype B epidemic in Japan. Considering limitation of understanding risk behaviors by interviews, in general, valid and reliable sexualities of individuals are difficult to obtain due to poor recall and embarrassment (26). Therefore, the transmission cluster analysis may compensate uncertainty of interview-based risk behaviors and establish consistency of collected information. In fact, most dTCs were predominantly male even though they included the heterosexual cases by the interview. Since MSM with subtype B is the most prevalent at-risk population since the 2000's in Japan (27), large dTCs often appeared to be MSM is not surprising. Then another question needs to be addressed, what transmission structure does the MSM population have?

The result implied that MSM communities in a geographic region are closed network and few interactions among the communities. This epidemiological situation is similar to that of other areas in high-income countries, where MSM has been significantly the at-risk population and transmission patterns were episodic (24, 28–32). Many local MSM groups in high-income countries commonly live within a city without substantial interaction between each other, and HIV is transmitted between MSMs along with the population structure. In contrast, transmission clusters of CRF01_AE infection in Japan were prevalent mainly among heterosexuals until the 2000's (10). Higher active transmission of subtype B in a local region than that of the CRF01_AE should have been not caused by the difference in the virological property but in the key population. In this context, recent increase of CRF01_AE viruses among MSM individuals (7) is another concern in Japan. The reports of domestically generated circulating recombinant forms of CRF01_AE viruses (33, 34) indicates that CRF01_AE viruses have invaded into Japanese MSM groups where subtype B viruses have spread, and the both subtypes were co-infected in an individual. In fact, CRF69_01B, one of such B-01 recombinants-related clusters, was identified as TC053 (*n* = 15). These conditions suggest that understanding the contribution of each MSM to TC formation and growth is increasingly important in establishing a prevention strategy.

MSMs was frequent in dTCs but were also found in singletons. What is the difference between MSMs who did and did not affiliate to dTC? The cases included in dTC were composed of younger (*p* = 0.007) and earlier diagnosed (*p* = 0.0064) MSMs (*p* = 0.0060) from middle-population regions (Tokai and Kyushu, *p* = 0.002). The number of young individuals slightly increased in dTC_≥10_ (Table 1B), suggesting that younger MSMs tend to gather in cliques. Because all samples were collected from ART naïve cases, the ratio of individuals in the earlier stage was consistent with higher CD4+ T-cell counts. This may lead to our observation that large dTCs showed a significantly higher average CD4+ T-cell count than the others. Taken together, MSMs in large dTCs may have been possible to take a HIV testing at earlier phase. Our finding also showed that two middle-population regions, Tokai and Kyushu, only associated with large dTC affiliation. The two regions include the third and fourth major cities in Japan, Nagoya and Fukuoka, respectively. The result suggests that developing a bigger dTC within a single region may require the optimal size of urban area. In general, MSM tends to migrate from rural areas or small cities to larger cities, where they can anonymously and easily engage in sexual encounters (35, 36). Therefore, larger cities will likely foster larger MSM groups. As gay community subculture is divided into various profiles based on the difference in trivial sexual behaviors (37), an urban MSM group is also subdivided based on their behavior. Some of them may sexually engage with their colleagues from other cities. Well-developed intercity train network, such as the super-express Shinkansen, might enhance the engagement. Contrary to Nagoya and Fukuoka that have excellent railway networks within the region, the Shinkansen has only intermediate or terminal stations. As a result, an infected MSM in a huge city might limitedly spread the virus within the local group in the meantime and then to other regions through an intercity network of MSMs. This may be a reason why the dTC affiliation rate in other studies objecting single big cities (24, 25) were much lower than our result. The optimal structure for TC development in a region implies that MSMs in the transmission network keep in close communication. Those networks members tend to be young and have early diagnosis, suggesting awareness on HIV infection of some young MSMs with a lot of intimate sexual friends. This is contrary to reports from high- and medium-income countries that young MSM did not undergo HIV testing (38, 39), but is consistent with the report that young MSM in Tokyo tended to visit hospitals during recent phase of infection (40).

The study has limitation that phylogeny-based identification of HIV potential transmission partners may not be identical with the real-world direct transmission link (41). Since the patients who participated in our drug resistance surveillance were recruited, selection bias may arise from both patients who choose hospitals outside our network and had not undergone HIV test. This may cause failure in finding the virus in the “missing link” that connects two dTCs that are originally one. dTCs identified here notably underestimated both the scope and number of MSM groups at risk for HIV. The selection bias also gives rise to the possibility of dTCs containing more females. Since male prevalence in the official HIV surveillance in Japan was 97% in 2012, and the number of cases with the available sequence was 22% of the total number reported, we assumed the male/female ratio in the sample to not be biased. The tendency of dTC to include early-diagnosed cases in young men may be an artifact, since a young person would obviously have a short time from infection to diagnosis due to the age factor.

In conclusion, many episodic MSM groups have simultaneously transmitted HIV-1 subtype B in Japan; some of which consisted of many and averagely young PLWHAs with recently diagnosed. The feature of those MSM groups varies based on the residential region as well as the preferred communication style of each group. This provides a clue in planning a preventive strategy tailored for potential high-risk populations. In this study, we found some sub-clusters in a large dTC as well as small dTCs consisting of the cases reported in a short period. If we target those clusters, we can expect the patients to have a high compatibility with active prevention programs. We also found some phylogenetic structures implying such a situation. On the other hand, PLWHAs who have not been tested will be in the unreachable dTCs. In this context, the few HIV-1 subtype B cases found in females in Japan may be due to the transmission to women via MSM, and not the other way around. Some men in Japan, despite being married to a woman, may continue MSM without her knowledge. Elimination of HIV stigma might allow recruitment of the unreachable MSM as well as relevant female partners in the tests. The future challenge is how to transport those patients who are likely concerned population in Japan (42, 43) to HIV testing.

## Data Availability Statement

The datasets presented in this study can be found in online repositories. The names of the repository/repositories and accession number(s) can be found at: AB640101-AB640604 and AB863746-AB871315.

## Ethics Statement

The studies involving human participants were reviewed and approved by the National Institute of Infectious Diseases and National Hospital Organization Nagoya Medical Center, Japan. The patients/participants provided their written informed consent to participate in this study.

## Author Contributions

TS, WS, and KY: conceptualization. JH and AH: study resources management. JH: sequence data deposition. TS: phylodynamic analysis and statistical analysis. JH and WS: BED assay. TS, JH, AH, and WS: writing, review and editing. WS and KY: funding acquisition. All authors contributed to the article and approved the submitted version.

## Conflict of Interest

JH is currently employed by the company MSD Japan. WS is currently employed by the company bioMerieux Japan. The remaining authors declared that the research was conducted in the absence of any commercial or financial relationships that could be construed as a potential conflict of interest.
