# Supplementary material for: Nation-Wide Viral Sequence Analysis of HIV-1 Subtype B Epidemic in 2003–2012 Revealed a Contribution of Men Who Have Sex With Men to the Transmission Cluster Formation and Growth in Japan
Source: Front Reprod Health. 2020 Dec 3;2:531212. doi: 10.3389/frph.2020.531212 (PMC9580810; doi:10.3389/frph.2020.531212)
Supplement: Supplementary file 1 [file Data_Sheet_1.docx]

Supplementary Material

# Supplementary Tables

**Supplemental Table 1. Demographic characteristics of the subtype B sample population.**

|  |  | Male | | Female | | Unknown | | Total | |
| --- | --- | --- | --- | --- | --- | --- | --- | --- | --- |
|  |  | n | % | n | % | n | % | n | % |
| Declared transmission risk | |  |  |  |  |  |  |  |  |
|  | Male-to-male sexual contact* | 2866 | 65.17% | 0 | 0.00% | 1 | 0.02% | 2867 | 65.19% |
|  | High-risk heterosexual contact | 381 | 8.66% | 54 | 1.23% | 0 | 0.00% | 435 | 9.89% |
|  | Intravenous drug user | 24 | 0.55% | 0 | 0.00% | 0 | 0.00% | 24 | 0.55% |
|  | Maternal to child transmission | 1 | 0.02% | 0 | 0.00% | 0 | 0.00% | 1 | 0.02% |
|  | Blood transfusion | 1 | 0.02% | 0 | 0.00% | 0 | 0.00% | 1 | 0.02% |
|  | Blood coagulation factor | 10 | 0.23% | 0 | 0.00% | 0 | 0.00% | 10 | 0.23% |
|  | Did not declared | 1034 | 23.51% | 6 | 0.14% | 20 | 0.45% | 1060 | 24.10% |
| Nationality | |  |  |  |  |  |  |  |  |
|  | Japanese | 3443 | 78.29% | 39 | 0.89% | 3 | 0.07% | 3485 | 79.24% |
|  | Asian countries | 50 | 1.14% | 4 | 0.09% | 0 | 0.00% | 54 | 1.23% |
|  | China | 11 | 0.25% | 0 | 0.00% | 0 | 0.00% | 11 | 0.25% |
|  | South Korea | 11 | 0.25% | 0 | 0.00% | 0 | 0.00% | 11 | 0.25% |
|  | North Korea | 1 | 0.02% | 0 | 0.00% | 0 | 0.00% | 1 | 0.02% |
|  | Chinese Taipei | 6 | 0.14% | 0 | 0.00% | 0 | 0.00% | 6 | 0.14% |
|  | Philippines | 6 | 0.14% | 1 | 0.02% | 0 | 0.00% | 7 | 0.16% |
|  | Malaysia | 2 | 0.05% | 0 | 0.00% | 0 | 0.00% | 2 | 0.05% |
|  | Indonesia | 3 | 0.07% | 0 | 0.00% | 0 | 0.00% | 3 | 0.07% |
|  | Thailand | 4 | 0.09% | 2 | 0.05% | 0 | 0.00% | 6 | 0.14% |
|  | Laos | 1 | 0.02% | 1 | 0.02% | 0 | 0.00% | 2 | 0.05% |
|  | Myanmar | 3 | 0.07% | 0 | 0.00% | 0 | 0.00% | 3 | 0.07% |
|  | Pakistan | 1 | 0.02% | 0 | 0.00% | 0 | 0.00% | 1 | 0.02% |
|  | Others | 1 | 0.02% | 0 | 0.00% | 0 | 0.00% | 1 | 0.02% |
|  | European countries | 11 | 0.25% | 0 | 0.00% | 0 | 0.00% | 11 | 0.25% |
|  | UK | 4 | 0.09% | 0 | 0.00% | 0 | 0.00% | 4 | 0.09% |
|  | France | 5 | 0.11% | 0 | 0.00% | 0 | 0.00% | 5 | 0.11% |
|  | Portugal | 1 | 0.02% | 0 | 0.00% | 0 | 0.00% | 1 | 0.02% |
|  | Russia | 1 | 0.02% | 0 | 0.00% | 0 | 0.00% | 1 | 0.02% |
|  | South American countries | 36 | 0.82% | 11 | 0.25% | 0 | 0.00% | 47 | 1.07% |
|  | Brazil | 19 | 0.43% | 10 | 0.23% | 0 | 0.00% | 29 | 0.66% |
|  | Peru | 15 | 0.34% | 0 | 0.00% | 0 | 0.00% | 15 | 0.34% |
|  | Argentina | 1 | 0.02% | 1 | 0.02% | 0 | 0.00% | 2 | 0.05% |
|  | Paraguay | 1 | 0.02% | 0 | 0.00% | 0 | 0.00% | 1 | 0.02% |
|  | North American countries | 26 | 0.59% | 0 | 0.00% | 0 | 0.00% | 26 | 0.59% |
|  | USA | 22 | 0.50% | 0 | 0.00% | 0 | 0.00% | 22 | 0.50% |
|  | Canada | 4 | 0.09% | 0 | 0.00% | 0 | 0.00% | 4 | 0.09% |
|  | African countries |  |  |  |  |  |  |  |  |
|  | Uganda | 0 | 0.00% | 2 | 0.05% | 0 | 0.00% | 2 | 0.05% |
|  | Oceanian countries | 4 | 0.09% | 0 | 0.00% | 0 | 0.00% | 4 | 0.09% |
|  | Australia | 3 | 0.07% | 0 | 0.00% | 0 | 0.00% | 3 | 0.07% |
|  | New Zealand | 1 | 0.02% | 0 | 0.00% | 0 | 0.00% | 1 | 0.02% |
|  | Unspecified foreigner | 23 | 0.52% | 0 | 0.00% | 0 | 0.00% | 23 | 0.52% |
|  | Unknown | 724 | 16.46% | 4 | 0.09% | 18 | 0.41% | 746 | 16.96% |
| Area of clinics and facilities | |  |  |  |  |  |  |  |  |
|  | Region 1 (Hokkaido) | 132 | 3.00% | 0 | 0.00% | 0 | 0.00% | 132 | 3.00% |
|  | Region 2 (Tohoku) | 57 | 1.30% | 1 | 0.02% | 0 | 0.00% | 58 | 1.32% |
|  | Region 3 (Kanto) | 2508 | 57.03% | 36 | 0.82% | 5 | 0.11% | 2549 | 57.96% |
|  | Region 4 (Koushinetsu) | 35 | 0.80% | 1 | 0.02% | 0 | 0.00% | 36 | 0.82% |
|  | Region 5 (Tokai) | 706 | 16.05% | 17 | 0.39% | 9 | 0.20% | 732 | 16.64% |
|  | Region 6 (Hokuriku) | 29 | 0.66% | 0 | 0.00% | 0 | 0.00% | 29 | 0.66% |
|  | Region 7 (Kinki) | 616 | 14.01% | 2 | 0.05% | 5 | 0.11% | 623 | 14.17% |
|  | Region 8 (Chugoku-Shikoku) | 27 | 0.61% | 0 | 0.00% | 0 | 0.00% | 27 | 0.61% |
|  | Region 9 (Kyushu) | 205 | 4.66% | 3 | 0.07% | 0 | 0.00% | 208 | 4.73% |
|  | Region 10 (Okinawa) | 2 | 0.05% | 0 | 0.00% | 0 | 0.00% | 2 | 0.05% |
|  | Unspecified | 0 | 0.00% | 0 | 0.00% | 2 | 0.05% | 2 | 0.05% |
| Age, years | |  |  |  |  |  |  |  |  |
|  | <20 | 32 | 0.73% | 1 | 0.02% | 0 | 0.00% | 33 | 0.75% |
|  | 20-29 | 931 | 21.17% | 10 | 0.23% | 0 | 0.00% | 941 | 21.40% |
|  | 30-39 | 1502 | 34.15% | 21 | 0.48% | 0 | 0.00% | 1523 | 34.63% |
|  | 40-49 | 724 | 16.46% | 13 | 0.30% | 0 | 0.00% | 737 | 16.76% |
|  | 50-59 | 331 | 7.53% | 5 | 0.11% | 0 | 0.00% | 336 | 7.64% |
|  | 60-69 | 159 | 3.62% | 4 | 0.09% | 0 | 0.00% | 163 | 3.71% |
|  | >70 | 43 | 0.98% | 1 | 0.02% | 0 | 0.00% | 44 | 1.00% |
|  | Unknown | 595 | 13.53% | 5 | 0.11% | 21 | 0.48% | 621 | 14.12% |
|  | Total | 4317 | 98.16% | 60 | 1.36% | 21 | 0.48% | 4398 |  |
|  |  |  |  |  |  |  |  |  |  |
| * Including bisexual male | |  |  |  |  |  |  |  |  |

**Supplemental Table 2. Maximum Likelihood fits of 24 different nucleotide substitution models.**

| Model | #Param | BIC | AICc | lnL | Invariant | Gamma | R |
| --- | --- | --- | --- | --- | --- | --- | --- |
| GTR+G+I | 961 | 61087.97391 | 50432.40624 | -24253.29332 | 0.450682368 | 0.622984298 | 4.187173048 |
| HKY+G+I | 957 | 61346.57205 | 50735.34054 | -24408.77634 | 0.453754593 | 0.619643529 | 4.253191529 |
| TN93+G+I | 958 | 61357.11168 | 50734.79611 | -24407.50016 | 0.452871494 | 0.609722354 | 4.195313191 |
| GTR+G | 960 | 61401.96356 | 50757.47992 | -24416.83413 | n/a | 0.296657466 | 4.190806855 |
| T92+G+I | 955 | 61444.75161 | 50855.68822 | -24470.95809 | 0.448474672 | 0.603408739 | 4.12431439 |
| K2+G+I | 954 | 61641.74001 | 51063.76069 | -24575.99828 | 0.444286278 | 0.579863695 | 3.955767365 |
| HKY+G | 956 | 61661.02073 | 51060.87327 | -24572.54666 | n/a | 0.290927692 | 4.269383239 |
| TN93+G | 957 | 61672.28946 | 51061.05794 | -24571.63504 | n/a | 0.28976501 | 4.327362001 |
| T92+G | 954 | 61757.05211 | 51179.07279 | -24633.65433 | n/a | 0.289976885 | 4.13457405 |
| K2+G | 953 | 61951.71712 | 51384.82189 | -24737.53282 | n/a | 0.285911949 | 3.975708746 |
| GTR+I | 960 | 63968.06434 | 53323.5807 | -25699.88452 | 0.520157325 | n/a | 3.916880617 |
| TN93+I | 957 | 64305.67031 | 53694.43879 | -25888.32546 | 0.520157325 | n/a | 3.926128763 |
| T92+I | 954 | 64307.72965 | 53729.75034 | -25908.9931 | 0.520157325 | n/a | 3.935477361 |
| HKY+I | 956 | 64333.88012 | 53733.73267 | -25908.97636 | 0.520157325 | n/a | 3.916060502 |
| K2+I | 953 | 64603.34321 | 54036.44797 | -26063.34586 | 0.520157325 | n/a | 3.794180493 |
| JC+G+I | 953 | 65336.19777 | 54769.30254 | -26429.77315 | 0.448207392 | 0.609010175 | 0.5 |
| JC+G | 952 | 65655.1213 | 55099.31016 | -26595.7809 | n/a | 0.303268141 | 0.5 |
| JC+I | 952 | 68232.29869 | 57676.48755 | -27884.36959 | 0.520157325 | n/a | 0.5 |
| GTR | 959 | 69061.48068 | 58428.08107 | -28253.13868 | n/a | n/a | 3.507763473 |
| T92 | 953 | 69351.33701 | 58784.44178 | -28437.34277 | n/a | n/a | 3.747135826 |
| TN93 | 956 | 69422.44424 | 58822.29678 | -28453.25842 | n/a | n/a | 3.773862272 |
| K2 | 952 | 69429.01806 | 58873.20692 | -28482.72928 | n/a | n/a | 3.734410925 |
| HKY | 955 | 69432.11276 | 58843.04937 | -28464.63867 | n/a | n/a | 3.774976252 |
| JC | 951 | 73035.7256 | 62490.99856 | -30292.62904 | n/a | n/a | 0.5 |

Models with the lowest BIC scores (Bayesian Information Criterion) are considered to describe the substitution pattern the best. For each model, AICc value (Akaike Information Criterion, corrected), Maximum Likelihood value (lnL), and the number of parameters (including branch lengths) are also presented. Non-uniformity of evolutionary rates among sites may be modeled by using a discrete Gamma distribution (+G) with 5 rate categories and by assuming that a certain fraction of sites is evolutionarily invariable (+I). Whenever applicable, estimates of gamma shape parameter and/or the estimated fraction of invariant sites are shown. Assumed or estimated values of transition/transversion bias (R) are shown for each model, as well. All positions with less than 75% site coverage were eliminated. That is, fewer than 25% alignment gaps, missing data, and ambiguous bases were allowed at any position. There were a total of 1017 positions in the final dataset.

**Supplemental. Table 3. Best models for each group of subtype B alignment set selecting by path sampling/stepping-stone sampling in Bayesian MCMC inference using BEAST**

| Group | The best models | | Log marginal likelihood | |
| --- | --- | --- | --- | --- |
|  | Clock | Demographic | Path Sampling | Stepping Stone |
| 1 | Strict | Exponential growth | -63862.724 | -63821.342 |
| 2 | Exponential | Constant size | -64113.921 | -64051.132 |
| 3 | Exponential | Constant size | -64023.143 | -64011.062 |
| 4 | Strict | Exponential growth | -63213.939 | -63209.842 |
| 5 | Strict | Exponential growth | -64014.382 | -64079.62 |
| 6 | Strict | Constant size | -63942.644 | -64001.671 |
| 7 | Strict | Exponential growth | -64118.635 | -64184.122 |
| 8 | Exponential | Constant size | -63978.431 | -64037.858 |
| 9 | Strict | Exponential growth | -64203.039 | -64281.301 |
| 10 | Strict | Constant size | -63901.801 | -63938.423 |
| 11 | Strict | Constant size | -63832.137 | -63912.722 |
| 12 | Strict | Constant size | -63783.941 | -63885.993 |
| 13 | Strict | Exponential growth | -63953.624 | -64023.977 |

Model selection for four molecular clock (strict, log-normal, exponential and random) and five demographic (constant, exponential, expansion, logistic and Bayesian Skyline Plot) models were performed in each phylogenetic group of subtype B cases by marginal likelihood estimation using path sampling and stepping-stone in BEAST. The best model for the inference were shown in each group.
